# It’s All in the Genes: The Regulatory Pathways of Sexual Reproduction in Filamentous Ascomycetes

**DOI:** 10.3390/genes10050330

**Published:** 2019-04-30

**Authors:** Andi M. Wilson, P. Markus Wilken, Magriet A. van der Nest, Michael J. Wingfield, Brenda D. Wingfield

**Affiliations:** Department of Biochemistry, Genetics and Microbiology, Forestry and Agricultural Biotechnology Institute (FABI), Faculty of Natural and Agricultural Sciences, University of Pretoria, Pretoria 0083, South Africa; markus.wilken@fabi.up.ac.za (P.M.W.); magriet.vandernest@fabi.up.ac.za (M.A.v.d.N.); mike.wingfield@fabi.up.ac.za (M.J.W.); brenda.wingfield@fabi.up.ac.za (B.D.W.)

**Keywords:** sexual reproduction, fungi, filamentous ascomycetes, gene expression, regulatory networks, functional characterisation

## Abstract

Sexual reproduction in filamentous ascomycete fungi results in the production of highly specialized sexual tissues, which arise from relatively simple, vegetative mycelia. This conversion takes place after the recognition of and response to a variety of exogenous and endogenous cues, and relies on very strictly regulated gene, protein, and metabolite pathways. This makes studying sexual development in fungi an interesting tool in which to study gene–gene, gene–protein, and protein–metabolite interactions. This review provides an overview of some of the most important genes involved in this process; from those involved in the conversion of mycelia into sexually-competent tissue, to those involved in the development of the ascomata, the asci, and ultimately, the ascospores.

## 1. Introduction

Fungi represent the most diverse of the eukaryotic kingdoms, with more than 100,000 species described to date [1]. While there is some disagreement regarding the actual number of fungal species present globally, this value is generally accepted to be in the millions [2,3]. Such a rich diversity has led to an incredible variety of life cycles as well as a high level of reproductive plasticity. As such, fungal species exhibit some of the most interesting mechanisms of propagation, both sexual and asexual [4].

**Figure 1:**
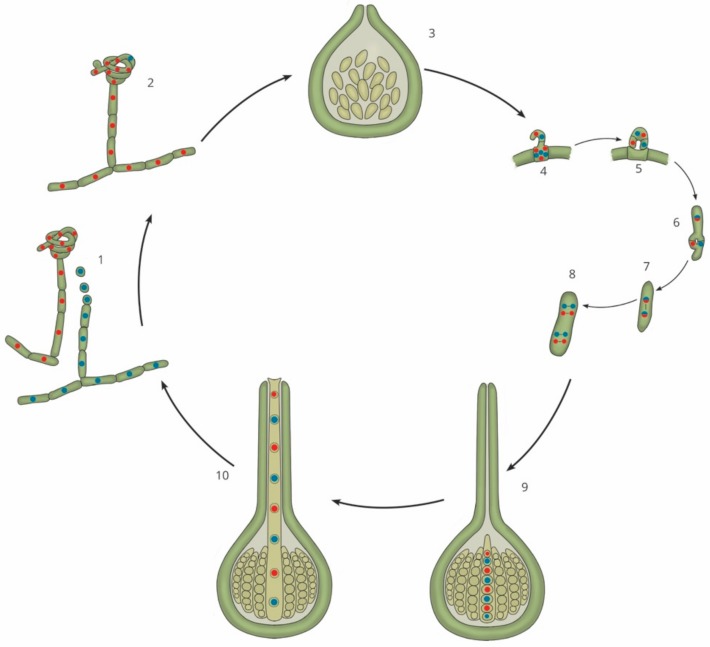
Generalized sexual cycle of filamentous ascomycetes. Mycelial strands (1) recognize a variety of signals before being converted into sexually competent tissue. One such signal is the recognition of a suitable mating partner. One partner produces the female structure, an ascogonium (indicated by the structure with red nuclei), while the second partner produces fertilizing spermatia (indicated by the single cells with blue nuclei). Gamete fertilization (2) occurs when the spermatia physically interact with the ascogonium. This is followed by the production of the protoascomata (3). Stages (4) to (8) occur within the immature ascomata and the developing asci. This includes the development of a crozier, nuclear migration, karyogamy, meiosis, and mitosis. This entire process culminates in a fully mature ascoma (9), which can release ascospores (10). These spores will then germinate and begin the cycle again.

Sexual reproduction in filamentous ascomycetes (Figure 1) is initiated upon the recognition of a variety of factors that cause the conversion of vegetative mycelia into sexually-competent tissue (reviewed in [5]). This is followed by gamete fertilization, and the production of a dikaryotic cell and the protoascomata, which mature into fully developed fruiting structures, known as ascomata. These structures harbour the sac-shaped asci that produce and protect the eight internal ascospores [6,7]. The production of these highly specialized sexual tissue types is a morphological outcome that relies upon the initiation and control of gene, protein, and secondary metabolite networks [8]. These networks interact with one another to ensure the correct spatiotemporal expression of gene products, which enables the process of sexual reproduction to take place under the most suitable environmental conditions.

In this review, we consider the many genes that play pivotal roles during the process of sexual reproduction in filamentous ascomycete fungi. To this end, three broadly divided sections are presented. These cover the major morphological changes that occur during protoascomatal development, ascus formation, and ascospore production.

## 2. Protoascomatal Development

The conversion of vegetative mycelia into sexually-competent, ascomatal-forming tissue is the first step in sexual reproduction. This relies on the recognition of various endogenous and exogenous cues, each of which uniquely contributes to whether sexual structures are produced.

### 2.1. The Mating Type Genes

**Figure 2:**
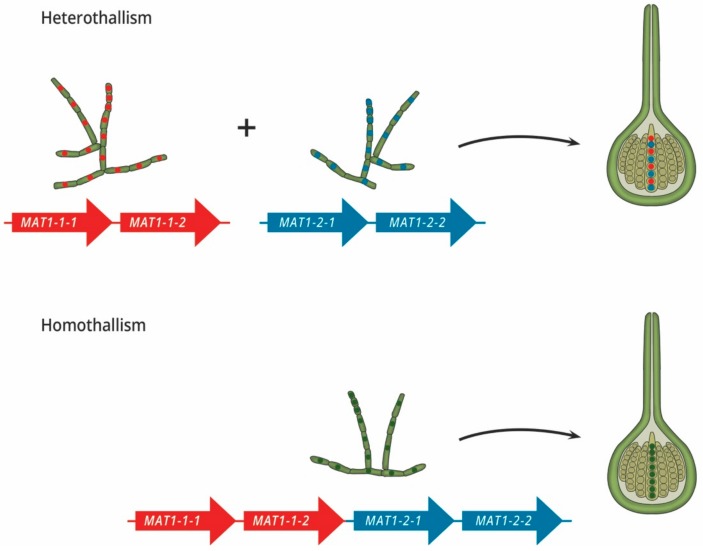
The sexual strategies of filamentous ascomycetes. Heterothallism: Two isolates of an opposite mating type need to physically interact in order to produce sexual structures. These mating types are genetically determined by genes at the *MAT* locus, either encoding the *MAT1-1* genes (red) or the *MAT1-2* genes (blue). These genes confer the MAT1-1 and MAT1-2 mating identities, respectively. Homothallism: Sexual reproduction can either occur within a single isolate that expresses both the *MAT1-1* and *MAT1-2* genes (as illustrated) or between any two individuals of the same species.

The master regulators of sex in most fungi are the mating type, or *MAT*, genes. These genes, harboured at the *MAT* locus, encode proteins that typically possess DNA binding domains [9]. The *MAT* proteins act as transcription factors that regulate the expression of genes related to sex, such as those involved in mate recognition, cellular differentiation, and meiosis [10,11]. It is the *MAT* genes that determine the mating strategies employed by a fungal species, as sexual reproduction typically requires the expression of *MAT1-1* and *MAT1-2* genes (Figure 2). In heterothallic fungi, individuals possess either the *MAT1-1* or *MAT1-2* genes, which confer the MAT1-1 and MAT1-2 mating types, respectively. This system requires opposite partners to physically interact in order to produce sexual offspring. In contrast, homothallic species typically possess both types of genes [12] and are thus able to sexually reproduce in complete isolate or with any other individual of the same species [4,13,14]. Until Turgeon and Yoder [15] proposed a revised naming system in 2000, *MAT* gene nomenclature was species-specific, making comparisons of gene content and function difficult. Thus, for the sake of this review, genes are referred to by the names that fulfil the requirements of the universal nomenclature system (Table 1), as revised and updated in 2017 by Wilken et al. [16].

In *Podospora anserina*, the sexual cycle is controlled by a variety of proteins that regulate the *MAT* genes. One of the most prominent *MAT*-regulating proteins is encoded by *pro1*, a gene that is very well characterized in a number of model fungi [17,18,19,20]. In *P. anserina*, this protein regulates similar pathways to those of other fungal species, but also plays an integral role in sexual reproduction [21]. *Pro1* acts as a positive regulator upstream of a high mobility group (HMG) box protein named PaHMG8, which itself positively regulates the expression of both *MAT1-1-1* and *MAT1-2-1* [21,22]. In addition to PaHMG8, two other HMG box proteins, PaHMG5 and PaHMG6, are also responsible for the tight regulation of the two *MAT* genes and their transcription networks [22]. Transcription factors that possess the HMG box domain are involved in a multitude of biological processes, with a particular importance in pathways associated with sexual reproduction in fungi [23].

Transcriptional control of the mating genes in *Trichoderma reesei* is achieved by a complex of proteins that respond to the availability of light [24,25]. In this species, two photoreceptors are encoded by the *blr1* and *blr2* genes, while a third regulatory protein is encoded by the *env1* gene [26,27,28]. Together, these three proteins regulate sexual reproduction in a mating-type dependent manner, by controlling the expression of *MAT1-2-1* [25]. The deletion of *env1* has a more pronounced effect on sexual reproduction than that of either of the photoreceptors, suggesting that BLR1 and BLR2 exert their effect via ENV1. Expression of *MAT1-2-1* is also controlled by VEL1, yet another protein involved in light-dependent sexual development in *T. reesei* [24].

The two primary *MAT* genes, *MAT1-1-1* and *MAT1-2-1*, have been functionally characterized in a variety of heterothallic and homothallic species. Unsurprisingly, both genes are typically essential for successful sexual reproduction, though their exact functions may differ from species to species. In *P. anserina*, *Aspergillus nidulans*, and *Neurospora crassa*, both genes are essential for fertilization and thus, while both female and male structures are often still produced in strains where either *MAT* gene has been deleted, further ascomatal development never takes place [11,29,30,31]. In addition to its role in fertilization, the *P. anserina MAT1-1-1* gene is also essential for ascospore production [29]. In *Fusarium graminearum*, both genes are essential and the deletion of either results in mycelia that is unable to form even immature sexual structures [32]. In *Penicillium chrysogenum*, the *MAT1-1-1* gene is not involved in the development of the ascoma, however, *ΔMAT1-1-1* strains do not produce ascospores and this gene is consequently essential for sexual reproduction [33]. The *P. chrysogenums MAT1-2-1*, however, has a much earlier role in sexual development as *ΔMAT1-2-1* strains do not form sexual structures, even in the presence of a suitable mating partner [34]. In contrast, *MAT1-1-1* is dispensable for sexual reproduction in *Sordaria macrospora*, while *MAT1-2-1* remains essential [35].

In addition to the primary *MAT* genes, other secondary genes have also been described from the *MAT* loci. Currently, six other *MAT1-1* genes (*MAT1-1-2* to *MAT1-1-7*) and 10 other *MAT1-2* genes (*MAT1-2-2* to *MAT1-2-11*) are known from various fungi (as reviewed in [16]). Although these genes often have no known functional domains and are not typically conserved beyond genus or family boundaries [12], a number of them have been at least partially characterized.

The secondary *MAT1-1* genes that have been shown to be essential to sexual reproduction include the *MAT1-1-2* genes of *S. macrospora* [10], *F. graminearum* [36], and *P. anserina* [37], as well as the *MAT1-1-5* genes of *Sclerotinia sclerotiorum* [38] and *Botrytis cinerea* [39]. Deletion of any of these genes results in the complete inability to produce sexual ascospores. While the exact function of the *S. macrospora* and *S. sclerotiorum* genes remain unknown, the *P. anserina MAT1-1-2* gene is directly responsible for ascus production, nuclear recognition, and cellular division [37,40]. The *F. graminearum MAT1-1-2* and the *B. cinerea MAT1-1-5* are both specifically involved in the development of the maturing protoascomata. Consequently, while mutant strains of both species produce immature sexual structures, asci-bearing ascomata never develop [36,39]. The *P. anserina MAT1-1-3* gene, although not essential for sexual reproduction, helps to determine the nuclear identity in sexually reproducing cultures [37]. Thus, this gene, in conjunction with *MAT1-1-1*, is responsible for nuclear recognition early in the sexual process, when the protoascoma is developing [37,41].

In addition to these *MAT1-1* genes, a variety of secondary *MAT1-2* gene are also essential for sexual reproduction. These include the *MAT1-2-4* genes of *S. sclerotiorum* [38] and *A. fumigatus* [42] as well as the *MAT1-2-10* gene of *B. cinerea* [39]. Interestingly, despite being homologs, the *MAT1-2-4* of *S. sclerotiorum* is important for ascomatal development [38], while the *A. fumigatus MAT1-2-4* gene appears to play a more global role in sexual reproduction by directly affecting the expression of a variety of different sex-related genes [42]. Lastly, the *B. cinerea MAT1-2-10* plays an important role in ascomatal development, with deletion strains unable to produce asci-harbouring fruiting bodies [39].

### 2.2. Important Signalling Pathways

One of the major protein complexes that control sexual development in fungi is the COP9 signalosome (CSN). This complex of proteins is involved in a huge diversity of processes because it plays an integral role in post-translational processes, including protein ubiquitination and phosphorylation [43]. In the most typical cases, the complex harbours up to eight individual subunits. One such example is encountered in *A. nidulans*, where genes *CsnA* through to *CsnH* are encoded by the genome [44]. The importance of this signalling pathway in sexual development is supported by the fact that mutant strains lacking the *CsnA*, *CsnB*, *CsnD*, and *CsnE* genes are unable to produce protoascomatal structures, despite initiating sexual development [44,45]. The deletion of a single *Csn* gene can affect the assembly of the entire signalosome, explaining the disrupted sexual development seen even in single gene knockouts [45].

The second important protein complex that is involved in sexual reproduction is known as STRIPAK, the striatin-interacting phosphatase and kinase complex (reviewed in [46]). This complex was first discovered in humans [47] and homologous complexes have since been identified in a huge variety of eukaryotic organisms, including *Drosophila melanogaster* [48]. The STRIPAK complex has been fairly well characterized in *S. macrospora*, where it is known to influence the production of the ascomata [49,50,51]. Three of the core proteins of this complex, PRO11, MOB3, and PRO22, have been studied in *S. macrospora* and are all essential for sexual reproduction.

The first of the STRIPAK genes to be characterized was *pro11*, which encodes a protein homologous to the mammalian striatin [51]. This gene is up-regulated during sexual reproduction [49], but is only involved in this process once the protoascomata have formed. Thus, while *Δpro11* mutants are sterile, ascogonia and immature ascomata are produced [51]. PRO11 interacts with a second protein, encoded by the *mob3* gene [49]. MOB3 is a phocein protein and like PRO11, is also up-regulated during sexual reproduction in *S. macrospora*. It is thus not surprising that *Δmob3* mutants are also sterile. However, the phenotypic disruption seen in *mob3* deletion mutants is more severe than that of *Δpro11* mutants, as they are unable to produce any sexual tissues [49]. The third protein, PRO22, interacts directly with PRO11 in the STRIPAK complex, and is involved in sexual development [50]. In *Δpro22* mutants, immature sexual tissue is formed, but does not develop further than the protoascomatal stage, which is similar to the *Δpro11* mutants [50,51].

### 2.3. Nutrient Requirements for the Induction of Sexual Reproduction

One of the most important sex-regulating factors that fungi are able to recognize and respond to is nutrient availability. Before sexual reproduction can occur, vegetative mycelia need to acquire nutrients that will support the energetically-intense process of sexual reproduction [52]. Nutrient availability is one of the environmental factors that influences the expression of the *MAT* genes [46]. In *P. anserina*, it is hypothesized that nutrient starvation activates *pro1*, which in turn regulates *PaHMG8*. As previously discussed, *PaHMG8* positively influences the expression of both *MAT1-1-1* and *MAT1-2-1*, thereby acting as an intermediate between nutrient availability signalling and the onset of sexual reproduction [21,53]. While the nutrient requirements for sexual reproduction differ amongst species, a few nutrients are essential to most. These are treated individually in the sections that follow.

#### 2.3.1. Sugars

Glucose and other sugars are the primary energy source for most living cells, including those of microorganisms [54]. Given that sexual reproduction is an energy-intensive process, carbon availability plays a significant role in its initiation. To this end, genes putatively encoding sugar-sensing proteins have been discovered in the genomes of many different fungi.

In *A. nidulans*, two of these proteins, *gprD* and *gprH*, have been partially characterized. The former shows significant similarity to the *Saccharomyces cerevisiae gpr1* [55] and the *Schizosaccharomyces pombe git3* [56] genes, both of which encode sugar sensing proteins. The latter, *gprH*, has been shown to recognize and respond to exogenous glucose levels [57]. Additionally, *ΔgprH* strains of *A. nidulans* undergo sexual reproduction without proper induction, while *ΔgprD* mutants display sexual reproduction under prohibitive conditions [57,58]. Furthermore, both mutants also show an increase in the expression of the mating pheromone receptors [57]. Taken collectively, these results indicate that the regulation of sex due to carbon availability is the result of a combined response by *gprD* and *gprH* [57].

The A. nidulans gprH in particular responds to periods of low carbon availability, in turn activating the cAMP-PKA (cyclic AMP- protein kinase A) pathway, an intracellular signalling pathway that ultimately initiates the transcriptional changes associated with the inhibition of sex [57]. During short-term starvation, *nosA*, a positive regulator of sex, is repressed [18,57]. This protein’s repression in turn activates a related protein, *rosA*, a negative regulator of sex [19,57]. Sexual reproduction is thus precluded until the stress associated with carbon starvation is alleviated.

#### 2.3.2. Amino Acids

**Figure 3:**
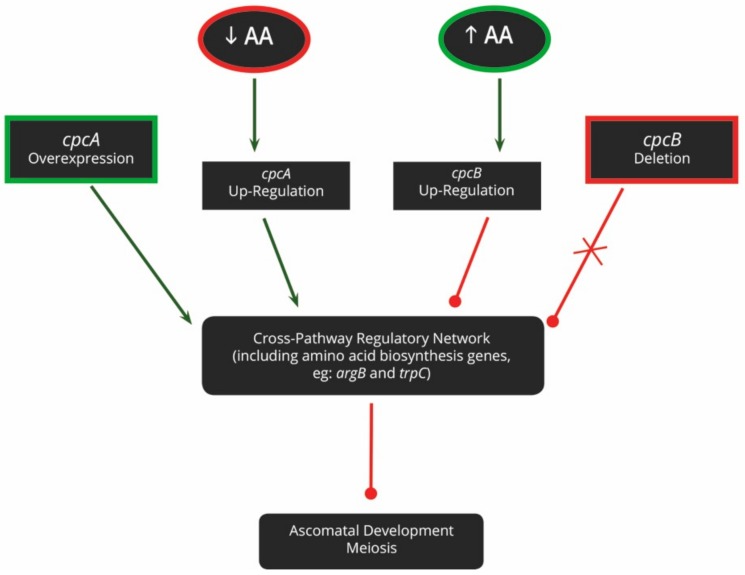
The involvement of the cross-pathway regulatory network (CPRN) during sexual reproduction in *A. nidulans*. In the absence of amino acids, *cpcA* is up-regulated, thereby activating the CPRN. This pathway subsequently inhibits the onset of sexual reproduction. In contrast, when amino acids are available, *cpcB* is up-regulated and the CPRN is inhibited. This results in sexual development. Either the overexpression of *cpcA* or the deletion of *cpcB* can activate the CPRN, by simulating amino acid deficiency, and thus also inhibit sexual reproduction. Shapes with green outlines indicate conditions under which sexual reproduction is directly or indirectly activated. Those with red outlines indicate conditions which directly or indirectly repress sexual reproduction. Green arrows indicate activation of a particular pathway, while red lines terminating in circles indicate repression.

Amino acids are crucial nutrients that determine whether sexual reproduction will take place (Figure 3). In *A. nidulans*, sensing and responding to exogenously available amino acids is achieved via the cross-pathway regulatory network (CPRN). This pathway integrates the amino acid biosynthesis pathways with other important cellular processes, such as sexual reproduction and virulence. The two major genes involved in this pathway are *cpcA* and *cpcB*, the cross-pathway control genes. While *cpcA* responds to amino acid deficiencies, *cpcB* recognizes exogenous amino acid availability [59].

The CPRN has been experimentally elucidated by generating a variety of mutants, including *cpcA* over-expression, *cpcB* deletion, and amino acid auxotrophic mutants. Both the *cpcA* and *cpcB* mutants experience amino acid starvation, regardless of the actual amino acid availability and consequently, sexual reproduction cannot take place [59,60]. These phenotypes can also not be rescued by the exogenous provision of amino acids, given their inability to recognize that amino acids are indeed available. In contrast, the phenotype of *ΔtrpA-D* auxotrophic mutants, which are unable to produce their own tryptophan and are thus sterile, can be rescued in the presence of an exogenous amino acid source [61,62]. Furthermore, *ΔargB* auxotrophic mutants are also sterile and produce only immature ascomata [63]. This phenotype can, however, not be rescued by an exogenous arginine supply, indicating that *argB* may play a greater role in sexual reproduction than simply aiding in the production of arginine.

#### 2.3.3. Calcium

As an essential element for most living organisms, calcium plays a variety of biological roles in the lives of many eukaryotes, especially during signal transduction [64,65]. To ensure consistent calcium availability, fungi utilize two different calcium uptake systems. The high-affinity calcium uptake system (HACS) functions when there are low levels of exogenous calcium and active uptake is required [66,67]. This system is discussed in more detail in the section dealing with ascospore discharge (see below). In contrast, the low-affinity calcium uptake system (LACS) is utilized when exogenous levels of calcium are high and active uptake is not necessary. This system is comprised of at least FIG1, a transmembrane calcium channel [68]. 

The LACS gene, *fig1*, has been extensively studied with regards to its role during sexual reproduction in *F. graminearum* [69,70], *A. nidulans* [71], and *N. crassa* [69]. Interestingly, while the importance of the gene is obvious in all three species, its actual function depends on the sexual strategy being employed by the species. For example, in both *F. graminearum* and *A. nidulans*, *fig1* is essential for the production of ascomata during homothallic mating [69,70,71]. However, *fig1* is completely dispensable in *A. nidulans* when an isolate undergoes outcrossing [71]. In contrast, the gene is important for heterothallic mating in *N. crassa*, particularly in MAT1-2 isolates, where *Δfig1* mutants are female sterile. Interestingly, this mutant phenotype is not seen in *Δfig1* MAT1-1 isolates, indicating a mating-type specific interaction [69].

### 2.4. Other Environmental Triggers

Once the critical nutrient and energy requirements have been met in the vegetative mycelium, a variety of other signals are needed to successfully initiate sexual reproduction.

#### 2.4.1. Light

Light can act as either a positive or negative regulator of sexual reproduction, depending on the species and the wavelength [72]. In order to respond to light-based signals, fungi express a variety of light-sensing molecules, which range from small peptides to giant protein complexes. This allows for the recognition of wavelengths across the visible light spectrum, from near-ultraviolet to red [72]. Two of the primary photo-recognition systems in fungi, the white collar system and the velvet complex system, have been intensively studied in *N. crassa* and *A. nidulans*, respectively.

In *N. crassa*, the blue light response relies on the recognition of blue light by WC-1, a white collar protein, which harbours the light-recognizing LOV (light oxygen voltage sensing) domain [73]. The WC-1 protein also harbours PAS (Per-Arnt-Sim) domains, which allows dimerization with both itself and a second white collar protein, WC-2 [74,75]. Additionally, both proteins possess zinc finger DNA binding motifs, which bind to the promoters of various light-inducible genes [75,76]. Thus, these domains allow the two proteins to form the entire signal transduction pathway associated with blue light illumination in *N. crassa*, from light recognition to transcriptional regulation.

Blue light enhances the production and development of ascomata in *N. crassa*. Thus, although not essential for protoascomatal production, illumination significantly increases sexual competency and the number of protoascomata produced [77]. Furthermore, upon fertilization and subsequent development, the ascomatal necks orient themselves towards the source of the blue light illumination [78], a form of phototropism that may act to enhance spore dispersal. Thus, while *Δwc1/2* mutants are not sexually defective, they produce far fewer protoascomata, most of which are unable to orient their necks correctly [77,78].

**Figure 4:**
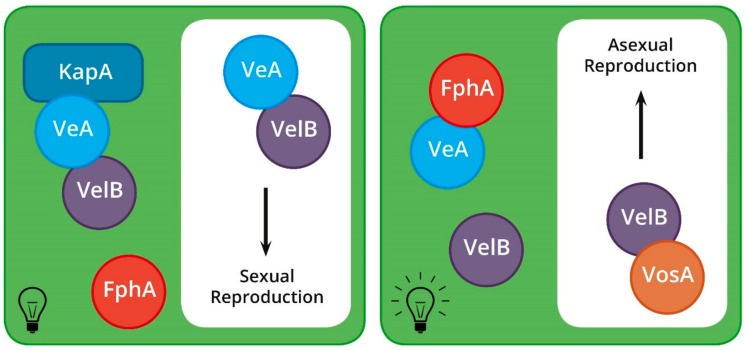
Sexual reproduction and light sensing in *A. nidulans*. Darkness: VeA and VelB interact with the importin protein, KapA. This allows their transport into the nucleus where they regulate gene expression and initiate sexual development. Light: FphA, the red light sensor, interacts with VeA, putatively inhibiting its interaction with KapA and VelB. This prevents the transport of VeA into the nucleus. Instead, VelB interacts with other proteins, activating asexual reproduction.

Light has a more direct effect on the sexual cycle of *A. nidulans* than that of *N. crassa* (Figure 4), with sexual reproduction being entirely precluded in the presence of light [79]. Furthermore, light sensing and its signal transduction pathway is more complex in *A. nidulans*, involving many proteins that form a variety of multi-peptide complexes [80,81]. The majority of these complexes are made up of the velvet proteins, which form different dimers and trimers during the *A. nidulans* life cycle, each of which elicits a unique response [81,82,83,84].

The first velvet gene, *veA*, is constitutively expressed in *A. nidulans* and displays a major up-regulation during sexual reproduction [82]. The gene is essential for mating, and, if overexpressed, leads to the production of ascomata under normally restrictive conditions [82]. A second velvet protein, VelB, which interacts with VeA in the cytoplasm, is also essential for sexual reproduction [81]. The VeA–VelB dimer interacts with a KapA importin protein during periods of darkness to bring about sexual reproduction [83]. The COP9 signalosome discussed above has also been shown to play an important role in regulating the recognition of light in *A. nidulans*. It is thought that this protein complex may be involved in the same pathway as *veA*, given that *ΔcsnD* mutants are unable to respond to light [45].

During periods of red light illumination, the light sensing phytochrome, FphA, is activated and binds to VeA, thereby preventing its transport into the nucleus [85,86]. Thus, *ΔfphA* strains are sexually competent even in the presence of light. However, *ΔfphA* mutants also produce significantly fewer ascomata than wild type isolates when grown in the dark [85]. This, in addition to the fact that FphA possesses kinase activity, indicates that the protein is most likely involved in additional functions not linked to light-sensing during sexual reproduction.

#### 2.4.2. Reactive Oxygen Species

Reactive oxygen species (ROS) are produced as by-products of aerobic respiration [87]. These radicals are known to cause damage to various cellular components, and thus most organisms express antioxidant systems for protection. The production of ROS is, however, increasingly being recognized as an important regulator of cellular processes, especially in cell differentiation and development [87,88].

In *A. nidulans*, genes involved in both ROS neutralization and production play integral roles in sexual development [89,90]. One such gene, *cpeA*, encodes a catalase-peroxidase that converts hydrogen peroxide into water. While *cpeA* is not expressed in vegetative mycelia, its expression dramatically increases during the onset of sexual reproduction [89]. It is hypothesized that *cpeA* is expressed as a mechanism to prevent ROS-induced damage to the developing fruiting structures. *A. nidulans* also expresses the ROS-producing enzyme NADPH oxidase [90]. This protein, encoded by *noxA*, produces superoxide molecules and is up-regulated in cultures induced to undergo sexual reproduction. The importance of ROS production in sexual reproduction has been illustrated by *ΔnoxA* mutants, which are unable to undergo homothallic mating, with sexual reproduction being blocked before protoascomata formation [90]. Interestingly, this knockout does not affect outcrossing, provided that the wild type partner can produce its own *noxA* and thus superoxide. It is thought that the ROS is responsible for signalling during cell differentiation prior to sexual reproduction and can be utilized by both interacting partners, regardless of which isolate produces it [90].

*N. crassa* expresses two NADPH oxidases that are essential for sexual reproduction. The first, *nox-1*, is a homolog of the *A. nidulans noxA* and is also significantly up-regulated during sexual development [91]. NOX-1 is specifically required for female fertility, and while *Δnox-1* strains are sexually incompetent if used as the female partner, these strains are able to fertilize wild type female structures [91]. *N. crassa* also expresses the *nox-2* gene, which, in contrast to *nox-1*, is highly expressed in asexual cells and prior to ascomatal development. This gene plays an important role in ascospore germination, despite the fact that *Δnox-2* strains produce normal sexual spores [91]. Although its exact role in germination is not understood, it is reasonable to speculate that the ROS produced by NOX-2 may be involved in a signalling pathway that initiates ascospore germination.

NADPH oxidases are also important regulators of sexual reproduction in *P. anserina*, both in early ascomatal production and later during ascus development and ascospore germination [53]. There are two NADPH oxidase encoding genes in the *P. anserina* genome, *nox1* and *nox2*. The two proteins are responsible for superoxide and peroxide secretion, a process which co-localizes with the developing protoascomata of sexually-competent isolates [53]. It is thought that *nox1* is responsible for a type of ROS-mediated signalling, which induces cell wall degradation of the surrounding cells and provides nutrients to the developing ascomata [53]. It is consequently not surprising that in *Δnox1* mutants, the number and size of protoascomata is greatly reduced. Furthermore, those that are fertilized take much longer to reach maturity than wild type protoascomata [53]. Given that a constant supply of fresh nutrients can rescue the mutant phenotype, the sexual defect is likely due to nutrient starvation.

#### 2.4.3. Pheromones

Pheromones are a broad class of chemical signals that are involved in the mating of species as diverse as mammals [92], insects [93], and reptiles [94]. These biologically active compounds are secreted into the environment and are recognized by an individual of the same species, but different gender, sex, or mating type [95]. Fungi utilize sex pheromones in the form of diffusible peptides that allow for mate recognition and attraction (extensively reviewed by [96,97]).

Genes encoding mating pheromones have been found in the genomes of many ascomycete fungi, including *Cryphonectria parasitica* [98], *Magnaporthe grisea* [99], *N. crassa* [100], *P. anserina* [101], and various *Fusarium* species [102]. As with the MAT genes, however, these genes have been given species-specific names. These genes mostly fall into one of two categories; those with similarity to the pheromone expressed by *S. cerevisiae* **a**-cells and those to the pheromone expressed by *S. cerevisiae* **α**-cells. For the purpose of this review, these pheromones will thus be termed the **a**- and **α**-factor pheromones, respectively (Table 1).

**Figure 5:**
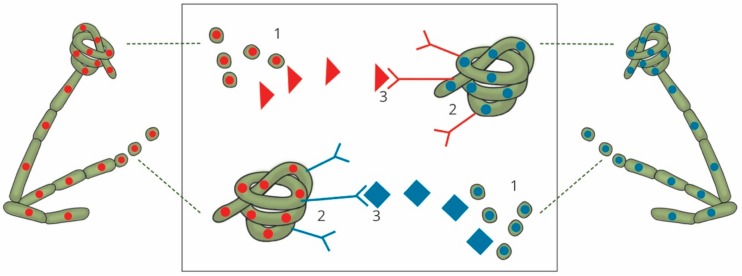
Pheromone signalling in heterothallic filamentous ascomycetes. (1) Pheromones are expressed, with spermatia of MAT1-1 isolates expressing the **α**-factor and spermatia of MAT1-2 isolates expressing the **a**-factor pheromone. (2) The ascogonia of these isolates also express the pheromone receptors, which recognize the pheromones. (3) Recognition of the pheromones by their receptors results in a variety of physiological changes, including growth towards the suitable partner as well as the transcriptional regulation of sex-related genes.

In many filamentous fungi, the **a**-factor and the **α**-factor pheromone genes are transcriptionally controlled by the *MAT* genes. Consequently, in heterothallic species, they are expressed in a mating-type dependent manner (Figure 5). In such species, the *MAT1-1-1* gene controls the expression of the **α**-factor, while the *MAT1-2-1* gene is responsible for **a**-factor expression. As a direct result, MAT1-1 individuals express only the **α**-factor and MAT1-2 individuals express only the **a**-factor [98,99,100,103]. Homothallic species, which typically possess both the *MAT1-1-1* and *MAT1-2-1* genes, are often able to express both pheromones [104,105].

Given that the pheromones are typically expressed by the male cells, either spermatia or conidia, it is not surprising that both pheromones play an essential role in the male fertility of *P. anserina* and *N. crassa* [101,106]. Deleting either of the factors results in isolates that cannot fertilize female isolates and are thus male sterile. Interestingly, while a complete knockout of the **a**-factor does not affect female fertility in *N. crassa*, disruption of the 3′ non-coding region greatly reduces protoascomatal production [107]. Additionally, the **α**-factor is also essential for male fertility in *C. parasitica* [108]. In contrast, the *C. parasitica* **a**-factor has been implicated in female fertility, where **a**-factor knockout strains produce only empty ascomata, but fully functional male structures [108,109]. Thus, while clearly important for the sexual process, the actual function of the pheromones differs slightly from species to species.

A third type of pheromone has been identified in certain heterothallic *Fusarium* species, as well as in the heterothallic *T. reesei* [102,110]. This pheromone harbours a number of repeating units as well as the terminal CaaX domain, and thus has characteristics of both the **α**- and **a**-factors, respectively. This hybrid pheromone has thus been termed the **h**-type factor [110]. Given its genomic location, it is thought that the gene encoding this factor assumed the function of the **a**-factor pheromone after the loss of the original **a**-factor-encoding gene. Furthermore, a typical **α**-factor pheromone gene has been found in the genomes of the species harbouring this **h**-type factor [102,110]. This illustrates the presence of a pheromone response pathway that, at least partially, resembles the typical **α**/**a** system. It is surprising, however, that the **α**-factor and **h**-type pheromones are not expressed in a mating-type dependant manner as seen in typically heterothallic species [110].

In order to recognise these pheromones, the female structures of these fungi express receptors that are able to specifically recognize each pheromone (Figure 5) [95]. These receptors belong to the class of seven transmembrane G-protein coupled receptors, which, when activated, initiate an MAP kinase signal transduction pathway (reviewed by [111]). Recognition of the pheromone by the receptor therefore initiates a transduction pathway, which in turn activates a variety of networks that lead to sexual reproduction. The pheromone receptors are also important regulators of pheromone expression and female fertility in *N. crassa*. Thus, the deletion of the **a**-factor receptor results in a decrease in **α**-factor expression in a MAT1-1 background [112] and deletion of the **α**-factor receptor results in the down-regulation of the **a**-factor in a MAT1-2 background [113]. Protoascomata are still produced by these mutants, indicating at least a partial activation of the female fertility pathway. However, these mutants are not able to recognize, grow towards, or fuse with fertilizing spores from a male isolate, thereby precluding successful fertilization and thus sexual reproduction [112,113].

The *F. graminearum* **α**-factor receptor also plays a role in female fertility [104]. Although it is not essential for sexual reproduction, female fertility is significantly reduced in gene deletion strains. Contrastingly, deletion of the **a**-factor receptor results in no observable sexual defects [104]. Given that *F. graminearum* is homothallic, it is perhaps not entirely surprising that a system with the primary function of mate seeking is no longer essential for sexual reproduction. This, however, is not true for all homothallic species, where expression of the pheromones and their receptors is indeed essential for the production of sexual spores [114,115,116]. 

Despite their apparent primary role in mate recognition, pheromones are hypothesized to play a role in some of the downstream processes associated with sexual reproduction [97,113]. This is substantiated by the fact that pheromone expression is not limited to heterothallic species, but is an important sex-promoting pathway in some homothallic species. Furthermore, the expression of pheromone pathway genes is not limited to the initiation of sexual reproduction and instead often continues throughout the entire process. Genes encoding both the pheromones and their receptors have been identified in the homothallic species, *S. macrospora* [105,115] and *F. graminearum* [104,117]. The genes encoding the **α**-factor pheromone as well as both the pheromone receptors have also been found in *A. nidulans* [118]. These genes are expressed and functional, despite the absence of mate seeking behaviour and are thus likely to have been co-opted into other functions.

In *F. graminearum*, the **α**-factor pheromone and its receptor are not essential for self-fertility, but single deletion mutants produce far fewer mature ascomata than wildtype isolates [104]. This indicates that this pheromone/receptor pair plays a role in ascomatal production or development. Unexpectedly, deletion of the **α**-factor gene also enhanced outcrossing events, indicating that **α**-factor expression may promote selfing, rather than outcrossing as in other species. This effect, however, is abolished if its receptor is also deleted [104].

Both pheromone receptor genes play an important role in homothallic mating in *A. nidulans* [116]. Deletion of either receptor results in a significant decrease in fertility, with mutants producing small ascomata that house a limited number of ascospores. Deletion of both the receptors results in an even more severe phenotype, where mutants are unable to produce ascomata at all [116]. Similar to *F. graminearum*, however, the disruption of either or both receptors does not affect outcrossing [116]. These genes are thus only important for selfing and play no role in outcrossing in this species.

In some species, pheromone expression has been proposed to facilitate post-fertilization events, particularly before karyogamy and meiosis in both homothallic and heterothallic species [119]. In *N. crassa*, the pheromones as well as their receptors are required for the production of the ascospores [107,113]. For example, forced mating interactions between a wild type isolate and an **a**-factor mutant result in the production of ascomata harbouring very few ascospores, while forced matings between mutants results in completely barren ascomata. Additionally, deletion of both a pheromone and its receptor in *N. crassa* results in protoascomata that never mature. Similarly, the deletion of the **α**-factor pheromone in *C. parasitica* results in the production of mature, but barren, ascomata [108,109]. Taken together, these results illustrate a role for both the pheromones and their response pathway in post-fertilization events, such as ascomatal, ascal, and ascospore development.

A combination of the above mentioned environmental and physiological factors ultimately lead to asexual reproduction, sexual development, or simply the continuation of vegetative growth. If all the requirements are met and sexual reproduction can take place, an immature fruiting body will form and develop into an ascoma. Subsequently, the asci can begin to form within the mature sexual structure.

## 3. Ascus Production

One of the defining characteristics of ascomycete fungi is the ascus, a sac-like structure in which the ascospores are produced and housed [6]. The wall of the ascus is unique compared to all other tissues formed by fungi, different even from others formed during sexual reproduction [120]. As such, there are a number of genes that are expressed almost exclusively by the ascus [121], ensuring that the most appropriate environment is created for the developing ascospores.

In *F. graminearum*, *amd1* is one such gene and is essential for the correct development of the ascus [120,122]. The gene encodes a transmembrane protein, which localizes to the ascus membrane and possesses a domain associated with transmembrane transport [122]. While the AMD1 protein has no apparent function in the production of normal ascomata, *Δamd1* strains are unable to produce stable ascus walls. Consequently, the wall degrades before the ascospores are ready for discharge, prompting their germination within the ascomata, and precluding effective dispersal [120,121,122]. Interestingly, the deletion of *amd1* results in the differential expression of many genes, including the up-regulation in membrane transport and the down-regulation of genes involved in cell-wall synthesis and cell-wall integrity [122]. This suggests that *amd1* is a master regulator of ascus wall synthesis and ensures cell wall integrity. This may be achieved by minimising cross-membrane transport and allowing the generation of turgor pressure in the ascus.

Ascus development genes have also been described from *N. crassa*, with functions mostly linked to gene regulation [123,124]. The ASD4 GATA DNA binding protein [123], the SMS-2 meiotic silencing Argonaute protein [125], and the STC1-like RNAi and chromatin remodelling protein [126] are all involved in regulating the production of the ascus. While ASD4 likely acts as a transcription factor, the other two proteins are intimately involved in the RNA silencing pathway [125,126]. The genes encoding these proteins are essential for this process, which is evident from the fact that mutant strains of *asd4*, *sms-2*, or *stc1*-like produce only empty ascomata that are incapable of forming asci. This extreme phenotype is thought to be due to the deregulation of the entire ascus development pathway, given the important role each protein plays in regulation [123,125,126].

In addition to its role in ascomatal development, ROS metabolism is also essential for ascus production in *P. anserina* [127]. The *car1* gene, now known as *pex2*, which encodes a peroxisomal assembly factor, ensures that peroxisomes are formed in abundance during asci maturation as well as ascospore delineation [127,128]. These peroxisomes play an important role in ensuring the sexual tissue is supplied with sufficient nutrients during sexual development [129]. In *pex2* mutants, these peroxisomes are not produced and the isolate is unable to undergo nuclear fusion prior to meiosis [127], precluding the development of the ascus. It is currently unclear whether this gene is essential for karyogamy itself, or whether it is involved in a process just upstream of nuclear fusion [129].

## 4. Ascospore Production

The production of ascospores represents the final step in sexual reproduction. If this process has been successful, these newly-produced spores will be discharged into the environment, where they germinate and initiate a new life cycle.

### 4.1. Meiosis and Ascospore Production

Ascosporegenesis is, by definition, dependant on a successful meiotic division cycle. There are a number of genes that have been identified in both *A. nidulans* and *N. crassa* that specifically ensure that prophase I, the first phase of meiosis, can begin. In *A. nidulans*, the deletion of either the *tubB* [130] or *grrA* [131] gene results in mutants that are able to produce asci-housing ascomata, but that cannot initiate meiosis. The similar phenotypes are particularly noteworthy given the different role each of these genes plays—while *tubB* encodes the structural α-tubulin protein, an important component in the microtubule assembly toolbox [130], *grrA* encodes a substrate adaptor protein that plays a role in the protein ubiquitylation and degradation pathway [131]. Similarly, deletion of either of the meiotic silencing genes, *sad1* and *sad2* of *N. crassa*, results in an almost identical phenotype, with mature fruiting bodies harbouring intact asci that are also unable to undergo meiosis [132,133].

Upon the completion of a successful cycle of meiosis, the developing ascospores must undergo further maturation, including partitioning and delineation within the ascus. In *N. crassa*, this process is at least partially regulated by *asd-1*, which encodes a rhamnogalacturonase that is expressed predominantly during mating [134]. In *Δasd-1* mutants, the eight nuclei formed during meiosis remain diffuse and do not delineate into individual spores, leading to the sterility of the mutants [134].

The maturation process depends on the expression of the cAMP-PKA pathway as well as correct calcium signalling in *F. graminearum*. Given the importance of both these pathways in signal transduction, it is not surprising that the proteins associated with these pathways are essential for sexual reproduction. CPK1, a catalytic subunit of the PKA, ensures the transition of an immature spore into a single-celled ascospore harbouring only one nucleus [135]. Additionally, the deletion of *mid1*, a component of the HACS (discussed below), results in abnormal ascospores that are two-celled and septate, with fragile cell walls [136].

### 4.2. Ascospore Discharge

Fungal spores tend not to be motile and thus many diverse and elegant mechanisms of propagule dissemination have evolved across the Kingdom. A common mechanism of dissemination includes the forcible discharge of ascospores into their environment. This typically involves a build-up of turgor pressure, followed by the swift release of the ascospores [137].

As discussed earlier in this review, calcium is an essential mineral required for sexual reproduction in certain filamentous fungi. While LACS was discussed in terms of ascomatal formation, HACS is important for the forcible discharge of ascospores in *F. graminearum* [70,136,137]. This supports the multi-phase importance for calcium during sexual reproduction, where this element is required during the initiation of sexual development as well as in the final stages of sexual reproduction.

The HACS is made up of two ion channels, *MID1*, a mechanosensitive protein [136], and *CCH1*, an L-type protein [137]. Mutant *F. graminearum* strains of both these genes are defective in their ability to discharge ascospores [70,136,137], with the mutant phenotypes being partially rescued by the provision of exogenous calcium. This potentially activates the LACS, allowing for calcium transport via another pathway. Interestingly, despite this obvious phenotype in *F. graminearum*, the *N. crassa mid1* homolog is dispensable for ascospore discharge and *mid1* deletion strains display no other sexual defects [138]. This provides an elegant example of how protein similarity, at the sequence or structure level, is not always a good predictor of shared function.

Ascospore release in *F. graminearum* also relies on the expression of a kinase gene, *kin1* [139]. This gene encodes a member of a kinase protein family that typically harbours proteins involved in both cell-polarity and microtubule transport [139,140]. The kinase localizes to the septal pores in the newly produced ascospores and subsequently to their germ tubes [139]. In knockout studies, *Δkin1* strains lack the ability to release the spores, despite having produced fully matured ascomata and asci [139]. Furthermore, the absence of *kin1* also interferes with the inhibition of ascospore germination prior to release. This results in the premature germination of the unreleased ascospores [139], similar to the phenotype previously discussed for *Δamd1* strains of the same species.

It is worth noting that not all ascomycetes forcibly discharge their spores. For example, in some species, including *Magnaporthe salvinii* [141] and those belonging to the polyphyletic grouping of the ophiostomatoid fungi [142], the asci deliquesce before the spores are discharged, thus leaving them free within the ascoma. Typically, these spores are then exuded in a slimy matrix from a pore, sometimes found at the tips of the ascomatal necks. Such fungi consequently rely on a variety of dissemination means, other than forcible discharge. In species of *Graphium, Dipodascus*, and *Ceratocystis*, for example, these exudates are typically sticky and can be picked up by insects, which then act as dispersal agents [143].

## 5. Conclusions

Sexual reproduction in fungi provides a complex, diverse, and intriguing system to study tissue differentiation in eukaryotes. Many fungi provide simple, easy to use models, often with very well-characterized life cycles. Given that sexual reproduction relies upon the recognition and response to a variety of endogenous and exogenous cues, this system can be used to model protein–protein, metabolite–protein, and protein–DNA interactions. Furthermore, signal transduction events as well as fine-scale and global changes in the transcriptome and proteome can be tracked in response to environmental changes.

Despite the intensive research that has already been committed to understanding the genes that play a role in fungal sexual reproduction, there are many important and intriguing questions that remain to be answered. Even in the best studied model organisms, certain aspects of sexual reproduction remain uncharacterized. This is partly due to the fact that many of the genes involved in sexual reproduction show pleiotropic effects when modified. For example, if they play a role early in sexual reproduction, this can preclude opportunities to understand how they function in later stages of the process as well. Furthermore, determining the role of genes in a single species does not necessarily imply that they have similar functions in other species, regardless of how closely related the species or how similar the gene sequence. Future research will thus likely be focused on identifying the downstream targets of the MAT transcription factors in different species. This will further elucidate the genetic pathways that underlie the different sexual strategies and identify the genes that ensure opposite mating type nuclei can be recognized as such.

The growing availability of next generation sequencing methods is facilitating increasingly rapid progress in many aspects of biological research. Genomics has, for example, allowed for the identification of genes involved in sexual reproduction in species previously thought to be asexual. Likewise, transcriptomics has enabled the elucidation of genes and pathways that are specifically expressed during sexual reproduction. In the future, we are likely to witness an increase in research that combines these technologies with classical functional characterization. This will be true not only for model species, but also for those fungi that are less well-known.

## Figures and Tables

**Table 1:** The gene name equivalents of the *MAT*, pheromone, and pheromone receptor genes.

Species	Sexual Strategy	*MAT1-1* Idiomorph	Universal Name	*MAT1-1* Genes	α-Factor Pheromone	α-Factor Receptor	*MAT1-2* Idiomorph	Universal Name	*MAT1-2* Genes	a-Factor Pheromone	a-Factor Receptor
*Cryphonectria parasitica*	Heterothallic	MAT1-1	*MAT1-1-1*	*MAT1-1-1*	*Mf1/1*	-	MAT1-2	*MAT1-2-1*	*MAT1-2-1*	*Mf2/1* *Mf2/2*	-
			*MAT1-1-2*	*MAT1-1-2*							
			*MAT1-1-3*	*MAT1-1-3*							
*Magnaporthe grisea*	Heterothallic	MAT1-1 ^a^	*MAT1-1-1*	*MAT1-1*	*MF2-1*	*ste3*-like	MAT1-2 ^a^	*MAT1-2-1*	*MAT1-2*	*MF1-1*	*ste2-like*
*Neurospora crassa*	Heterothallic	mat A	*MAT1-1-1*	*matA-1*	*ccg4*	*pre2*	mat a	*MAT1-2-1* *MAT1-2-2*	*mata-1* *mata-2*	*mfa-1*	*pre1*
			*MAT1-1-2*	*matA-2*							
			*MAT1-1-3*	*matA-3*							
*Trichoderma reesei*	Heterothallic	MAT1-1	*MAT1-1-1*	*mat1-1-1*	*ppg1* ^c^	*pre2*	MAT1-2	*MAT1-2-1*	*mat1-2-1*	*hpp1* ^c^	*pre1*
			*MAT1-1-2*	*mat1-1-2*							
			*MAT1-1-3*	*mat1-1-3*							
*Podospora anserina*	Pseudo-homothallic	mat-	*MAT1-1-1*	*FMR1*	*mfm*	*pre2*	mat+	*MAT1-2-1*	*FPR1*	*mfp*	*pre1*
			*MAT1-1-2*	*SMR1*							
			*MAT1-1-3*	*SMR2*							
*Sordaria macrospora*	Homothallic ^b^	mat A	*MAT1-1-1*	*SmtA-1*	*ppg1*	*pre2*	mat a	*MAT1-2-1*	*Smta-1*	*ppg2*	*pre1*
			*MAT1-1-2*	*SmtA-2*							
			*MAT1-1-3*	*SmtA-3*							
*Aspergillus nidulans*	Homothallic ^b^	MAT-1	*MAT1-1-1*	*MAT1-1*	*ppgA*	*preB*	MAT-2	*MAT1-2-1*	*MAT2-1*	-	*preA*
*Fusarium graminearum*	Homothallic ^b^	MAT1-1	*MAT1-1-1*	*MAT1-1-1*	*ppg1*	*pre2*	MAT1-2	*MAT1-2-1*	*MAT1-2-1*	*ppg2*	*pre1*
			*MAT1-1-2*	*MAT1-1-2*							

^a^ Prior to the revised nomenclature of mating type genes, the *MAT* loci of *Magnaporthe* spp. were arbitrarily named Mat1-2 and Mat1-1. While these names have since been changed to comply with the naming system, the pheromone gene names still reflect the original *MAT* gene names. ^b^ In homothallic fungi, the mating type genes are all found within a single genome. Thus, it is not necessary to classify the locus as *MAT1-1* or *MAT1-2*. However, these species do still harbour *MAT* genes with homology to those found in heterothallic species. ^c^ *T. reesei* harbours the **h**-type pheromone in place of the **a**-factor pheromone found in many other species. Furthermore, the two pheromones of *T. reesei* are not expressed in a mating-type dependant manner. This is covered in the section regarding pheromones.
